# Hierarchical Emulsion-Templated Monoliths (polyHIPEs) as Scaffolds for Covalent Immobilization of *P. acidilactici*

**DOI:** 10.3390/polym15081862

**Published:** 2023-04-13

**Authors:** Zhengqiao Yin, Shengmiao Zhang, Xiucai Liu

**Affiliations:** Shanghai Key Laboratory of Advanced Polymeric Materials, Key Laboratory for Ultrafine Materials of Ministry of Education, School of Materials Science and Engineering, East China University of Science and Technology, Shanghai 200237, China; zhengqiaoyin12138@163.com

**Keywords:** cell immobilization, high internal phase emulsion, polyHIPE, fermentation, _L_-lactic acid

## Abstract

The immobilized cell fermentation technique (IMCF) has gained immense popularity in recent years due to its capacity to enhance metabolic efficiency, cell stability, and product separation during fermentation. Porous carriers used as cell immobilization facilitate mass transfer and isolate the cells from an adverse external environment, thus accelerating cell growth and metabolism. However, creating a cell-immobilized porous carrier that guarantees both mechanical strength and cell stability remains challenging. Herein, templated by water-in-oil (w/o) high internal phase emulsions (HIPE), we established a tunable open-cell polymeric P(St-*co*-GMA) monolith as a scaffold for the efficient immobilization of *Pediococcus acidilactici* (*P. acidilactici)*. The porous framework’s mechanical property was substantially improved by incorporating the styrene monomer and cross-linker divinylbenzene (DVB) in the HIPE’s external phase, while the epoxy groups on glycidyl methacrylate (GMA) supply anchoring sites for *P. acidilactici*, securing the immobilization to the inner wall surface of the void. For the fermentation of immobilized *P. acidilactici*, the polyHIPEs permit efficient mass transfer, which increases along with increased interconnectivity of the monolith, resulting in higher _L_-lactic acid yield compared to that of suspended cells with an increase of 17%. The relative _L_-lactic acid production is constantly maintained above 92.9% of their initial relative production after 10 cycles, exhibiting both its great cycling stability and the durability of the material structure. Furthermore, the procedure during recycle batch also simplifies downstream separation operations.

## 1. Introduction

Biorefining research and exploration has consistently been one of the cutting-edge techniques as a substituting strategy for exhaustible fossil resources in light of the rising environmental challenges of fossil fuel overuse [1,2,3,4,5,6]. Microbial fermentation, as an essential component of biorefinery engineering, provides sustainable bioenergy or chemicals by using natural biomass resources as ingredients to produce green chemical raw materials, promoting social and economic sustainability, and adhering to current carbon neutrality [7,8]. Despite a wide range of applications, the inherent issues of conventional microbial fermentation systems, such as environmental blockage, product inhibition, and cell elution, have always been detrimental to fermentation output and efficiency.

Therefore, developing an appropriate delivery system is critical to protect sensitive cells from adverse environments and improve metabolic product yield. The immobilized cell fermentation technique (IMCF) aims to constrain the cells to the water-insoluble carrier that supports cell growth and metabolism. This IMCF system can be desirable over freely suspended cells since immobilization normally promotes an improvement in metabolic performance, cell stability, and product separation during fermentation, as well as permitting repeated catalysis for adherent cells [9,10]. In IMCF, it is crucial to establish an applicable carrier or scaffold that facilitates cell growth and the metabolism process since the surface chemistry of the immobilized carrier material can affect the activity and stability of immobilized cells [11,12]. Diverse carriers have recently been developed for cell immobilization to achieve excellent mass transfer and long-term cell viability. P. S. Panesar et al. established a calcium pectate gel immobilized system to entrapped *Lactobacillus casei* for _L_ (+) lactic acid production from whey, and the optimized pectate-entrapped bacterial cells achieved a high lactose conversion and corking reusing ability [13]. Despite the most frequently utilized matrix in IMCF, natural materials such as Ca-alginate and pectin are solely focused on mass transfer enhancement and cell biocompatibility. Due to their low mechanical strength and susceptibility to corroded corrosion, natural carriers cannot retain stability against agitation and shear. To provide sufficient mechanical properties, inorganic mesoporous material was developed as support for the advantages of large specific surface area, narrow void distribution, and good resistance against biodegradation [14,15,16]. Zijian Zhao and colleagues absorbed *Lactobacillus rhamnosus (L. rhamnosus*) in mesoporous silica-based carriers under mild conditions and the Isi-*L. rhamnosus* exhibited excellent reusability for lactic acid production due to its stabilization against disruption [17]. However, this attachment approach could not efficiently capture cells and is easy to cause leakage of already immobilized cells throughout the fermentation process due to the weak binding effect. Mechanical strength in highly stable cell-carrier systems can also be enhanced through the use of other strategies, such as synthetic polymer and composite materials. Fabricio dos Santos Belgrano et al. designed a 3D-printed porous nylon bead and modified the microbead with polycationic polyetherimide (PEI) [18]. Such microbeads are very stable against stirring and acidic conditions, and the cell-bound microbeads may be used as inoculants even after extended storage. Nevertheless, the cells could not adhere to the 3D-printed nylon bead lack of PEI better; conversely, treatment of the beads with PEI decreased productivity due to the polycation’s inhibitory action. It is still tough to immobilize cells in a polymeric porous carrier that is both mechanically robust and highly active in the culture medium environment.

Recently, high internal phase emulsion (HIPE) templated porous polymeric material (polyHIPE) has been built as a promising scaffold for cell immobilization owing to its well-defined and fully interconnected microporous structure [19,20,21,22]. PolyHIPE is prepared by solidifying the continuous phase of a HIPE (i.e., an emulsion with its internal phase occupying at least 74.05 vol.%), which provides significant benefits over other porous material fabrication due to its exceptional flexibility and controllable structure [23,24,25,26,27,28]. The liquid flow in the hierarchical void structure of polyHIPE complies with Murray’s law, and the microchannels between adjacent voids not only preserve its structural integrity but also boost mass transfer efficiency during cellular metabolism [29,30]. Furthermore, more stable cell adhesion may be achieved by the surface modification of void walls, such as functionalized group modification or adding functional polymeric monomers. Despite its 3D-scaffold, polyHIPE has demonstrated rapid cell adhesion ability and excellent biocompatibility for cell growth in IMCF [31,32,33,34,35]; in the practically applied fermenter, the mechanical qualities of certain polymerization materials, such as polyglycolide acid (PGA), are usually insufficient for the fermentation. The mechanical strength of polyHIPE can be controlled by both porous structures and selected monomers in the continuous phase, which has great potential for applications in the culture and fermentation of immobilized microbial cells.

In this study, we described a hierarchically polymeric monolith as a scaffold for the efficient immobilization of microbial cells. As a case study, an engineered cell of *P. acidilactici* was immobilized onto mechanically sound polyHIPE to convert glucose to lactic acid in a recycled-batch fermentation process. Initially, the w/o HIPE was prepared by homogenizing the CaCl_2_ aqueous solution into the organic solution composed of styrene (St), divinylbenzene (DVB), and glycidyl methacrylate (GMA). Then the HIPE template polyHIPE was synthesized by copolymerizing the external phase monomer, and the obtained P(St-*co*-GMA) polyHIPE was used to covalently immobilize the cells for fermentation. The used engineered microbial cell of *P. acidilactici* completely and coordinately converted the glucose into high-titer and high-charity _L_-lactic acid [36]. The aim of this study was to test the feasibility of overcoming the bioprocess limitations by the use of a novel scaffold designed and produced by polyHIPE as a support for microbial cells. The monomer of St was employed to enhance the mechanical property, and the effect of void size, interconnectivity, and functionalization with GMA on cell adhesion and fermentation was studied.

## 2. Results and Discussion

### 2.1. Preparation and Characterization of Immobilized polyHIPE Scaffold

Herein, a P(St-*co*-GMA) polyHIPE monolith was prepared via an emulsion-templating method for microbial cell immobilization. Prior to the templating copolymerization, a w/o HIPE was prepared by homogenizing the oil and water with a high internal phase fraction. The polyHIPE was synthesized by curing the external phase of the HIPE template, in which the St functioned as a mechanical monomer, whereas GMA was employed as a functional monomer to immobilize cells covalently. Moreover, the incorporation of the cross-linker DVB could improve polyHIPE’s structural stability [37]. The polyHIPE was characterized by FT-IR. As shown in Figure S1, the strong absorption peak at 2924 cm^−1^ is attributed to the C-H stretching vibration of the -CH_2_- structure in copolymers, whereas the peak at 1734 cm^−1^ is associated with the C=O group in GMA. The ring vibrations of the benzene ring were in the absorption bands of 1601, 1493, and 1452 cm^−1^, and the bending vibration of hydrogen atoms on the mono-substituted benzene ring absorbs at 1029 and 759 cm^−1^, respectively. The characteristic peak of the epoxy group of GMA is present at 907 cm^−1^. The absorption peaks at 797, 839 cm^−1^ are indicative of dibasic substituted compounds at the adjacent and interposition of the DVB benzene rings, which is approximately similar to the spectra in the literature, demonstrating the successful modification of functional group GMA on the monolith [38].

**Figure 1:**
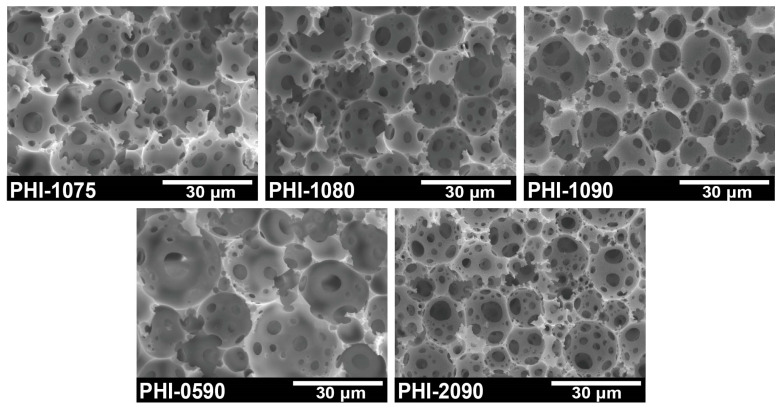
SEM images of open-cell polyHIPE scaffolds.

The obtained polyHIPE was supposed to be applied to immobilize cells, and the structure must be figured out as the first factor that the monolith should be set up to facilitate the movement of cells within the polyHIPE scaffold. As seen in Figure 1, all generated polyHIPEs have an open-cell void structure, and it can be observed that the void structure of polyHIPE varies with different emulsifier Span80 concentrations or varied internal phase ratios. As is summarized in Table 1, it could be seen that the average void size reduced from 18.9 μm to 11.6 μm as emulsifier concentration increased from 5 wt.% to 20 wt.%. In addition, along with the internal phase fraction increasing from 75 vol.% to 90 vol.%, the void size decreased from 16.1 μm to 12.5 μm. This finding reveals that the void size of polyHIPE can be tuned by appropriately changing the parameters for the preparation of HIPE. In IMCF, the size of the pore throats should be larger than the size of the bacteria so that the cells can enter the voids without resistance before being completely immobilized, and the size of the voids should be large enough to provide the cells for metabolism and growth activities. It can be seen from Table 1 that the size of the pore throats tends to be a similar level (3–4 μm), which is sufficient for the entering of *P. acidilactici* to fulfill their admission requirements. Moreover, despite the variations, the void sizes of the entire given polyHIPEs are highly satisfying and provide a spacious site for the cells. 

**Figure 2:**
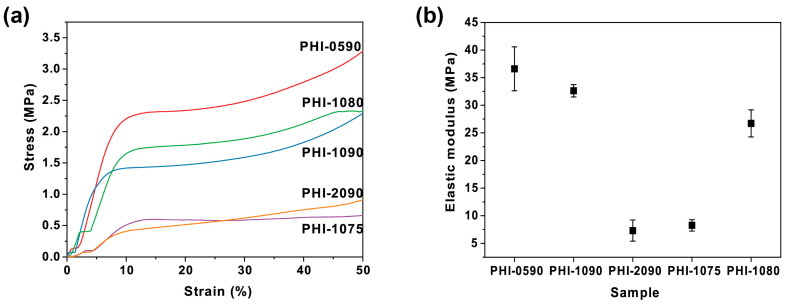
Stress–strain curves (**a**) and Young’s modulus (**b**) of the varied polyHIPE scaffolds under compressive load.

When the polyHIPEs are used for cell immobilization and subsequent fermentation, the cells and broth infiltrate the polyHIPEs, and the capillary effect acts on the scaffold [39,40]. Moreover, since the polyHIPE is constantly shaken in the broth throughout the fermentation, it would be eroded by the broth or collide with the container walls, which requires the scaffold to have significant mechanical strengths to preserve its intrinsic porous structure against the complex fermentation process. The mechanical properties of the polyHIPEs herein were characterized by measuring the compressive strength (Figure 2). Figure 2a depicted a typical polyHIPE stress–strain curve, with a linear region at low strains, a brief stress plateau, and a densification stage with a sharp increase in stress [41]. Attribute to the polystyrene skeletal frame and the stiff cross-linker DVB, the polyHIPE scaffolds exhibit excellent mechanical properties with a high Young’s modulus (*E*). In detail, as is shown in Figure 2b, by comparing Young’s modulus of PHI-0590 (36.6 MPa), PHI-1090 (32.6 MPa), and PHI-2090 (7.3 MPa), a decrease in Young’s modulus was displayed with the increase in the amount of Span80 used in the preparation of the emulsion. Moreover, the polyHIPE with increasing internal phase fraction had an enhanced Young’s modulus (*E*_PHI-1075_ = 8.3 MPa, *E*_PHI-1080_ = 26.7 MPa, and *E*_PHI-1090_ = 32.6 MPa). It is worth mentioning that the Young’s modulus of given polyHIPEs in this study exceeds that of most scaffolds widely used in cell culture today, such as PVA-based materials (*E* = 1.88 MPa) and F127-BUM gel (*E* = 0.67 Mpa) [42,43], and its robust mechanical property could ensure the structural integrity during the process of subsequent immobilization and several cycles of fermentation.

The smooth flow of substrate inside the material is very important, i.e., the interconnectivity of the porous material should be sufficient to ensure a sufficient mass transfer rate. As is shown in Table 1, the interconnectivity of polyHIPEs increased along with the volume ratio of the internal phase increasing (PHI-1075, PHI-1080, and PHI-1090). Moreover, because the emulsifier could reduce the HIPE droplet size and increase the interconnectivity of porous materials [27,44], the interconnectivity was improved as the Span80 content increased (PHI-0590, PHI-1090, and PHI-2090).

**Figure 3:**
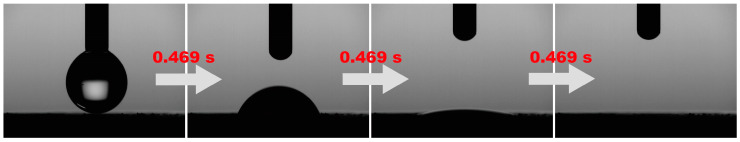
Water wettability of PHI-1090 scaffold.

Given that the medium is a kind of aqueous solution, the wettability of the material is a crucial prerequisite for effective following cell entrance and mass transfer. The water wettability of the polyHIPE was then investigated by putting a drop of water onto the sample. As shown in Figure 3, taking PHI-1090 as an example, when roughly 20 μL of water is dropped upon a sample, the water permeates the monolith within 1.5 s. Great water wettability was also proved by other polyHIPEs (PHI-0590, PHI-2090, PHI-1075, and PHI-1080) with infiltration time within 2 s for a drop of water (Figure S2), and the wetting rate increased with an increase in the interconnectivity of polyHIPE. The favorable water permeability ensures a smooth mass transfer during subsequent cell immobilization and fermentation.

### 2.2. Immobilization Process

The cell immobilization procedure is divided into two phases. Firstly, *P. acidilactici* cells were collected by centrifuging the seed broth at 4 °C and distributed in a PBS buffer (20 mM, pH = 7.4), which aimed to remove the culture medium and limit further cellular growth and proliferation. Then 1 g of sterilized polyHIPE scaffold was placed in the above PBS dispersion of *P. acidilactici*, and shaken at 150 rpm at room temperature for cell immobilization, in which the cells were attached to the carrier of the porous scaffold. During this process, due to the high reactivity and plasticity of the epoxy group, which on GMA provided anchoring points for cells on the void wall and reacted directly with the -NH_2_ group on the cell surface for covalent immobilization [45].

**Figure 4:**
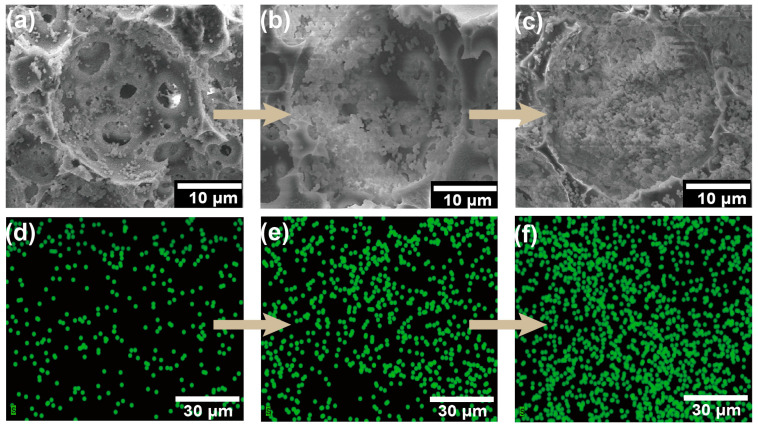
SEM images of *P. acidilactici* immobilized PHI-1090 (**a**), *P. acidilactici* immobilized PHI-1090 after one cycle fermentation (**b**), and *P. acidilactici* immobilized PHI-1090 after 10 cycle batches (**c**). (**d**–**f**) were sulfur element EDS images, corresponding to (**a**–**c**), sequentially.

The immobilized *P. acidilactici* was characterized via SEM-EDS images. Before observation, the immobilized cells were treated using 2.5 wt.% glutaraldehyde as a fixative to freeze the cell morphology. The cells were subsequently dehydrated using an ethanol gradient before the cell-immobilized polyHIPE was freeze-dried. SEM-EDS images of the distribution of cells within the voids are shown in Figure 4a,d. Figure 4a shows that the cells were located on the void walls, which demonstrated that the *P. acidilactici* was successfully immobilized on the polyHIPE support. The successful immobilization of *P. acidilactici* was further confirmed by the sulfur element EDS mapping as the sulfur element in sulfhydryl groups is only present in cells in this system (Figure 4d).

The quantitative analysis of the immobilized *P. acidilactici* was investigated by determining nitrogen contents with elemental analysis; the nitrogen mass fraction in the *P. acidilactici* was maintained at 11.4 wt.%, which can be used as a reference standard to figure out the content of immobilized bacteria, which was calculated to 0.04 g/g (the immobilized efficiency was 75.3 wt.% compared to the cells wight before immobilization), which demonstrated the excellent characteristics of the polyHIPE on the immobilized cells. Since the cells are covalently immobilized on the PHI, the cells are firmly bound to the scaffold and not easily dislodged. In contrast to the cells adsorbed on the carrier [46], the initial cell amount on PHI can be kept constant. Moreover, as cells were immobilized on the carrier, the material’s hydrophilicity rose, resulting in an increase in water wettability and permeability (Figure S3).

### 2.3. Fermentation Process of Immobilized P. acidilactici

**Figure 5:**
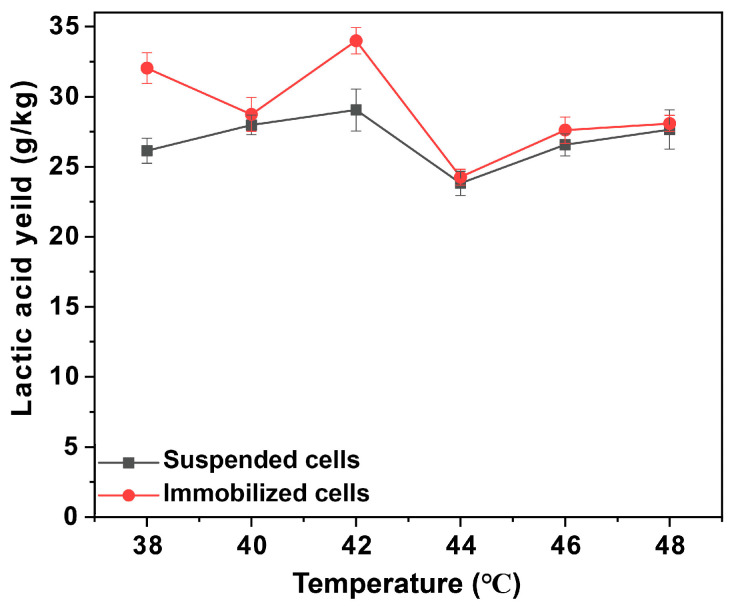
The _L_-lactic acid concentration for *P. acidilactici* immobilized PHI-1090 fermented at different temperatures.

The fermentation of lactic acid by immobilized *P. acidilactici* was conducted under an anaerobic environment. The distribution of cells in the voids at the end of fermentation can be observed by SEM-EDS mapping (Figure 4b,e), from which it can be seen that the number of cells in the voids multiplied substantially, proving the extraordinary biocompatibility of the polyHIPE scaffold. Then the optimum temperature of the immobilized *P. acidilactici* fermentation was characterized by the lactic acid production fermented under a series of temperature gradients. As shown in Figure S4, the ^1^H-NMR spectrogram showed double peaks at δ = 1.10–1.11 ppm, which are characteristic peaks of _L_-lactic acid. Take PHI-1090 as an example, as shown in Figure 5 and Table S1, in the temperature range of 38–48 °C, the _L_-lactic acid produced by the immobilized cells was higher than that of the suspended cells owing to the low thermal conductivity of the polyHIPE. In addition, 28.6% of the remaining activity of the immobilized cells was retained at 40 h, which is higher than alginate immobilized cells (55% at 4 h) [47], demonstrating the excellent temperature stability of the PHI immobilized cells. The optical purity of _L_-lactic acid produced by immobilized *P. acidilactici* was 99.6 ± 0.1%, which was equivalent to that of _L_-lactic acid generated by suspended *P. acidilactici*, indicating that immobilization had no influence on the _L_-lactic acid generation. 

**Figure 6:**
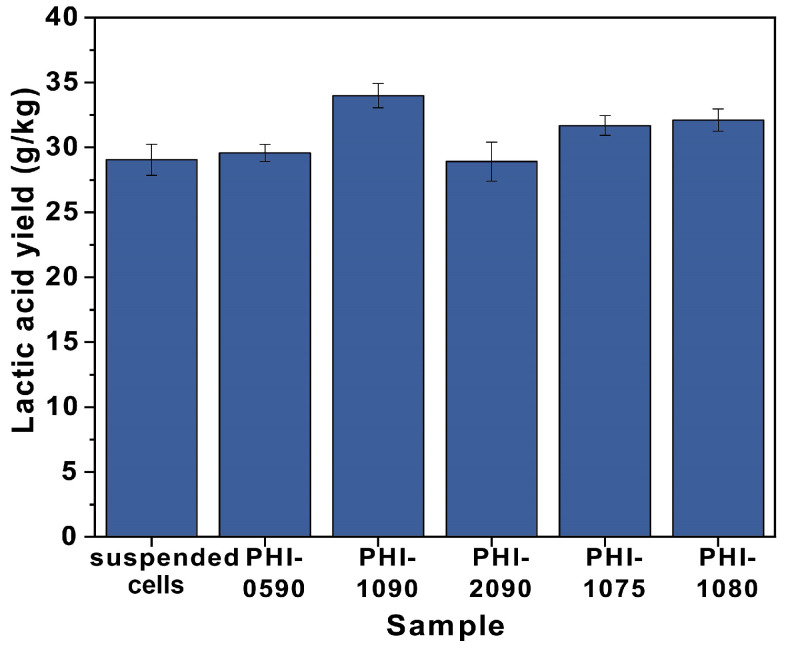
The _L_-lactic acid concentration for suspended and immobilized *P. acidilactici* on different PolyHIPE scaffolds at 42 °C.

**Figure 7:**
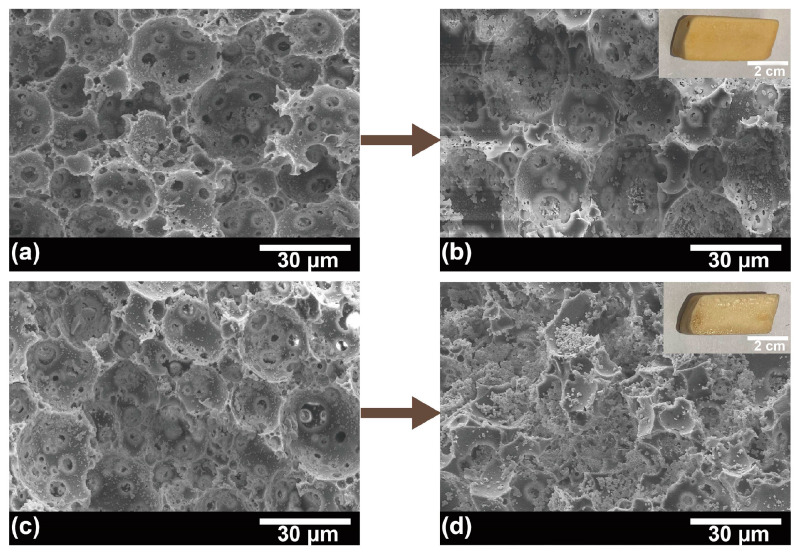
SEM images of *P. acidilactici* immobilized PHI-1090 before (**a**) and after one fermentation (**b**). Cell immobilized PHI-2090 before and after fermentation are shown in (**c**) and (**d**), respectively. The insets in (**b**,**d**) are the corresponding PHI photographs.

The structural dependency of _L_-lactic acid production at 42 °C by immobilized cells was evaluated by immobilizing *P. acidilactici* on various structural carriers. As shown in Figure 6 and Table S2, the lactic acid yields of the immobilized cells were all higher than those of the free cells, with a maximum yield increase of 17.6%. Moreover, by comparing the _L_-lactic acid yield from the fermentation of *P. acidilactici* immobilized PHI-1075, PHI-1080, and PHI-1090, the lactic acid yield improved as the polyHIPE interconnectivity increased, which is because higher interconnectivity leads to a more efficient mass transfer. This result can also be seen from PHI-0580, and PHI-1090, where the highest yield of lactic acid was obtained after PHI-1090 fermentation, and the morphology of the PHI-1090 after undergoing a fermentation process could be seen in Figure 7a,b. However, *P. acidilactici* immobilized on PHI-2090 had the highest interconnectivity but lower fermentation efficiency, which may be caused by the lower mechanical properties of PHI-2090, and the shaking during fermentation may damage the porous structure, resulting in low lactic acid yield (Figure 7c,d).

**Figure 8:**
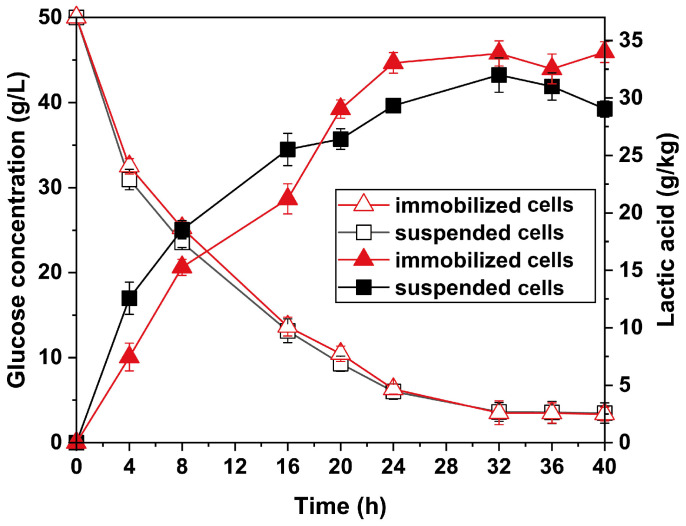
The concentration of glucose to _L_-lactic acid yield for suspended and PHI-1090 immobilizing *P. acidilactici* within 40 h.

The fermentation process occurred when the monolith of immobilized *P. acidilactici* PHI-1090 was introduced directly to the fermentation medium at 42 °C to convert glucose to _L_-lactic acid within 40 h. The cell capacity in the support was measured by 0.11 g/g after 40 h, which was much higher than the primary content (0.04 g/g), indicating cell growth during fermentation. The glucose and _L_-lactic acid concentrations during fermentation were monitored. As shown in Figure 8, the glucose was consumed from 50 g/L to 3.5 g/L and 3.4 g/L for suspended and immobilized *P. acidilactici*, respectively. In addition, the generated _L_-lactic acid concentration was 34.0 g/kg, with an increase of 17% compared to that of suspended cells (29.1 g/kg), exceeding a lot of reported lactic acid yield enhancements by immobilized cell fermentation (Table S3) and demonstrating that the immobilized cells of *P. acidilactici* by PHI could effectively utilize glucose to produce _L_-lactic acid [17,48,49,50]. Interestingly, the _L_-lactic acid production of the suspended cells was initially higher than that of the immobilized cells, but from 20 h the immobilized cells produced more _L_-lactic acid compared to the suspended cells. This phenomenon could be explained as follows. Firstly, cells of *P. acidilactici* tend to form biofilms on the void walls, which are preferable to typical biotransformation of suspended cells [51]. On the other hand, during immobilized cell fermentation, as the number of cells rises, the diffusion of oxygen and nutrients inside the void limits cell growth, resulting in increased _L_-lactic acid yields owing to the utilization of glucose in a _L_-lactic acid generation [48,52].

### 2.4. Recyclability and Reusability of Immobilized P. acidilactici

**Figure 9:**
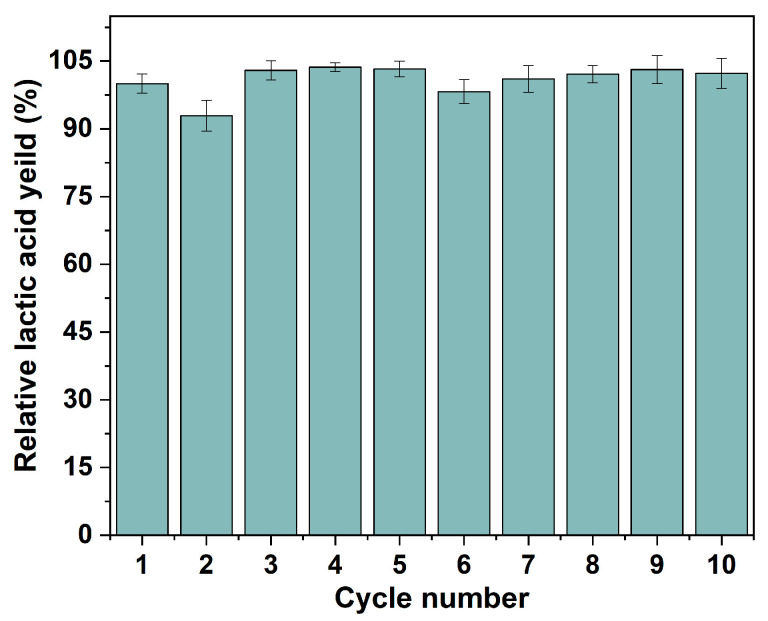
Recyclability and reusability of *P. acidilactici* immobilized PHI-1090 scaffold.

One of the greatest advantages of the IMCF is the cells’ reusability to be reused following medium change. As shown in Figure 9, the relative yield of lactic acid was consistently stable over 92.9% in 10 cycles, which is higher than that of free cells (80.8%, Table S4). In addition, the immobilized *P. acidilactici* displays higher lactic acid yield in each cycle compared to suspended cells, reflecting the immobilized *P. acidilactici* herein is greatly recyclable and reusable. The cell content in the polyHIPE support was 0.17 g/g measured by an elemental analyzer after 10 batch fermentations. This reveals that the fermentation process was carried out with a significant cell proliferation in the voids, and the effective mass transfer during the fermentation process. Moreover, corresponding to Figure 4c, the physical resistance and the existence of the immobilized *P. acidilactici* after multiple batches were confirmed by the SEM-EDS. During the recycling batch, the enhanced amount of cells in the polyHIPE could be seen in Figure 4f, which was primarily responsible for the synthesis of lactic acid [53]. Both the macro-morphology of the polyHIPE and the microstructure of the hierarchical void are relatively well maintained under continuous violent shaking during repeated fermentation, indicating the excellent mechanical properties and thus excellent cell retention of the polyHIPE (Figure S5). Furthermore, the *P. acidilactici* immobilized PHI-1090 was placed in a fermenter for amplified fermentation (Figure S6), and showed equal fermentation cyclability.

## 3. Experimental Section

### 3.1. Materials

Styrene (St, 99%, Macklin Co., Ltd., Shanghai, China), glycidyl methacrylate (GMA, 97%, Macklin Co., Ltd., Shanghai, China), and divinylbenzene (DVB, 80%, Aladdin Co., Ltd., Shanghai, China) were purified by filtration through basic and neutral alumina three times to remove the inhibitor before use. Sorbitan monooleate (Span80, HLB = 4.3, CP, Macklin Co., Ltd., Shanghai, China) and calcium chloride (CaCl_2_, 99%, Aladdin Co., Ltd., Shanghai, China) were used as received. Azobisisobutyronitrile (AIBN, 98%, Shanghai Titan Scientific Chemical Regent Co., Ltd., Shanghai, China) was recrystallized twice and dried before use. Glutaraldehyde (25 wt.%, Macklin Co., Ltd., Shanghai, China) was diluted 10 times before use. Ethanol (99.5%, Aladdin Co., Ltd., Shanghai, China) was diluted gradient to 30%, 50%, 70%, and 90% before use. PBS buffer (20 mM, pH = 7.4) was prepared in the lab and 3,5-dinitrosalicylic acid (DNS) reagent was prepared in the lab and stored in a brown vial. Sterilized deionized water was used.

*P. acidilactici* (CGMCC #13611) is the engineered lab lactic acid bacterium for _L_-lactic acid production with inserted xylose assimilation pathways [36]. The cells were lyophilized and stored in a refrigerator at −20 °C. Before immobilization, the cells were added to 50 mL of seed medium of de Man, Rogosa and Sharpe (MRS) medium and were cultured for 16 h at 42 °C. The MRS medium consists of 20 g/L of glucose (99%, Macklin Co., Ltd., Shanghai, China), 10 g/L of peptone (BR, Solarbio Science & Technology Co., Ltd., Beijing, China), 10 g/L of yeast exact (Yuanye Bio-Technology Co., Ltd., Shanghai, China), 5 g/L of sodium acetate (99%, Shanghai Titan Scientific Chemical Regent Co., Ltd.), 2 g/L of diammonium citrate (98%, Macklin Co., Ltd., Shanghai, China), 2 g/L of dipotassium hydrogenphosphate (99.9%, Macklin Co., Ltd., Shanghai, China), 0.58 g/L of magnesium sulfate heptahydrate (99.9%), and 0.25 g/L of manganese sulfate monohydrate (99.9%, Aladdin Biochemical Technology Co., Ltd., Shanghai, China) [54]. The pH value of the seed medium was 5.5, and the seed medium was shaken at 150 rpm.

### 3.2. Method

#### 3.2.1. Preparation of polyHIPE Scaffold

St (1.8 g), GMA (0.2 g), DVB (0.9 g), and Span80 (0.39 g, 10 wt% relative to monomers) were added to a 100 mL beaker and stirred until well mixed as an oil phase before the initiator AIBN (0.12 mmol) dissolved. Then, 10.4 mL of deionized water (80 vol.%) with CaCl_2_ was added to the oil phase at room temperature, and the mixture was homogenized at 400 rpm. The resulting emulsion was transferred into a 10 mm × 10 mm × 15 mm PTFE mold and cured for 10 h at 70 °C. The prepared polyHIPE was washed with ethanol and then deionized water to remove unreacted monomers and leftover Span80. The given monolith, which was called PHI-1080, was dried and then sterilized in an autoclave for 20 min at 121 °C.

In order to investigate the different polyHIPE carrier structures, other polyHIPEs prepared by different amounts of Span80 and aqueous phase fraction were also prepared in the same way, which can be seen in Table 1.

#### 3.2.2. Immobilization Process

The seed culture solution was centrifuged in a cryogenic centrifuge (4 °C) with a speed of 4000 rpm. The separated *P. acidilactici* (67.0 mg) was washed with PBS buffer before it was dispersed in a 50 mL PBS buffer. Then 1.0 g of the carrier was added to the cell dispersion of PBS solution and shaken under 150 rpm for 24 h at room temperature. After immobilization, the obtained cell-immobilized carrier was rinsed with sterilized deionized water three times to remove the suspended cells.

#### 3.2.3. Fermentation Process of Immobilized *P. acidilactici*

The optimum temperature of immobilized *P. acidilactici* for _L_-lactic acid fermentation under an anaerobic environment was first determined. A total of 1 g of *P. acidilactici* immobilized polyHIPEs were placed in the fermentation medium in a 250 mL conical flask with 50 mL simplified MRS medium as fresh fermentation broth containing 50 g/L glucose, and shaken under 150 rpm for 40 h at 38, 40, 42, 44, 46, and 48 °C, respectively, and the _L_-lactic acid concentration was measured by ^1^H-NMR. Then immobilized cell polyHIPEs were used for fermentation at the optimum temperature to further find the optimum fermentation parameters. PolyHIPEs with varying structures were obtained by adjusting the content of Span80 and the volume fraction content of the HIPEs’ internal phase (see Table 1 for details on these parameters) and were used to immobilize the cells and subsequently conduct fermentation to find the highest lactic acid yield. The found *P. acidilactici* immobilized polyHIPE with optimum parameter was utilized for fermentation and the fermentation process was monitored for the _L_-lactic acid yield and glucose consumption by withdrawing 500 μL of the broth every 4 h. Other fermentation conditions remained the same as before.

The free cell fermentation as a control experiment was conducted as follows. Firstly, 5 mL (10 vol.%) of seed medium was withdrawn and added into a 50 mL fermentation medium containing 50 g/L glucose, and the fermentation was conducted by shaking under 150 rpm for 40 h. A total of 500 μL of the broth was withdrawn every 4 h and the substrate and product content was measured.

#### 3.2.4. Recyclability and Reusability of Immobilized *P. acidilactici*

The recyclability of the *P. acidilactici*-loaded polyHIPE was investigated via repeat-batch fermentation for 400 h (10 batch cycles) at 42 °C. Following the completion of each batch (i.e., 40 h), the fermentation broth was removed and substituted with a freshly prepared medium. Collected immobilized *P. acidilactici* polyHIPEs were rinsed in sterilized deionized water three times (20 min for once) between batches and then re-introduced to a 50 mL of fresh fermentation medium for the next fermentation loop. The free cell reusability was conducted by withdrawing 10 vol.% of fermentation broth of each batch and transferring it into a fresh medium for the next fermentation cycle.

#### 3.2.5. Characterization

FT-IR spectrum of the P(St-*co*-GMA) based polyHIPE scaffold was characterized using an FTIR spectrometer (Nicolet 6700).

The microstructure of polyHIPEs and cell-immobilized polyHIPEs were observed by scanning electron microscopy (SEM, S-4800, Hitachi, Japan). In order to estimate the mean size of the voids and interconnected pores, 100 voids in the SEM image were investigated using the software Image J. For the cell observation, the cell immobilized polyHIPE was soaked into 2.5 wt.% of glutaraldehyde for 40 min at 35 °C, and then the sample was transferred and submerged for 15 min in a gradient concentration of ethanol. The interconnectivity (I) of the polyHIPE was calculated according to Equation (1):

(1) $$\;\mathrm{Interconnectivity}\;\left(I\right)=\frac{1}{4}n\;\times \;{\left(\frac{d}{D}\right)}^{2}\times 100$$

where n is the average number of interconnected pores per void. D and d are the average diameters of the void and interconnected pores, respectively.

For the mechanical strength test, the compression moduli of polyHIPEs were measured as follows: a cube sample of about 10 mm × 10 mm × 10 mm was made and squeezed with a WDW-50E universal testing machine (Sansi, Shenzhen, China) at a constant rate of 1.0 mm/min until half the height of the sample was reached. The stress–strain curve was used to compute the Young’s modulus (*E*) from the slope of the curve’s initial linear region, and it is the average value obtained by compressing three samples.

The material’s wettability was characterized using a contact angle goniometer (JC2000C Contact Angle Meter, Powereach Co., Shanghai, China). A drop of water (about 20 μL) was dripped onto the sample. The contact angle was determined by averaging the results of five measurements taken at various locations on the same sample.

The qualitative analysis of immobilized cells was characterized by an energy dispersive spectrometer (EDS, QUANTAX 400-30), which evaluates the location of microbial cells within the carrier by analyzing the sulfur element distribution of the material microregion components.

The quantitative analysis of immobilized cell content in the polyHIPE was determined by an elemental analyzer (Elementar Vario EL Cube). Simultaneously, quantitative analysis was conducted on the control sample of polyHIPE carriers without cells. The cell content within the support is determined by calibrating the pure cells before immobilization for elemental nitrogen content, and the immobilized capacity (g/g) was calculated according to Equation (2).

(2) $$\mathrm{Immobilized}\;\mathrm{capacity}\;(g/g)=\frac{\omega_{\mathrm{im}}-\omega_{\mathrm{polyHIPE}}}{\omega_{c}}$$

where ω_im_ (wt.%) and ω_c_ (wt.%) are the mass fractions of elemental nitrogen of cell immobilized polyHIPE and pure cells, respectively. ω_polyHIPE_ (wt.%) is the mass fraction of elemental nitrogen in the polyHIPE without cell and is used to correct for testing errors of the elemental analysis instrument.

The Megazyme _D_-/_L_-Lactic Acid Kit was utilized in order to conduct an analysis of the chiral purity of the _L_-lactic acid (Megazyme International Ireland, Bray, Wicklow, Ireland). 

The reduced glucose was determined using the 3,5-dinitrosalicylic acid (DNS) assay [55,56,57]. The standard curve was obtained as follows: Take 0 mL, 0.2 mL, 0.4 mL, 0.6 mL, 0.8 mL, and 1.0 mL of glucose standard solution (1.0 mg·ml^−1^) in a 15 mL tube, fill to 1.0 mL with distilled water, and add 2.0 mL of DNS reagent, respectively. The solutions were heated in a boiling water bath for 2 min, cooled with running water, and fixed with water to a 15 mL scale. The absorbance was measured at 540 nm using a UV-5800 spectrometer (Shanghai Metash Instruments Co, Ltd., Shanghai, China). For sample determination, the sample was diluted appropriately to make the sugar concentration within the range of 0.2–0.6 mg·ml^−1^. A total of 1.0 mL of the diluted sugar solution was added in a 15 mL tube, and boiled for 2 min before 2.0 mL of DNS reagent was added. The mixture was cooled down and filled up with water to a 15 mL scale, and measured the absorbance. The glucose content was obtained from the standard curve. 

The product _L_-lactic acid was determined by ^1^H-NMR (AVANCEIII 500 MHz spectrometer, Bruker, Germany). A total of 1.0 mL of the post-fermentation mixture was taken and centrifugated at 10000 rpm. A total of 500 µL of the supernatant was withdrawn and mixed with 100 μL of internal standard solvent dimethyl sulfoxide-d6 (DMSO-d6) for a ^1^H-NMR test.

## 4. Conclusions

In this study, we have designed a mechanically robust hierarchical monolith as a cell scaffold for the immobilization and recycling batch fermentation of *P. acidilactici* to generate _L_-lactic acid. First, highly ordered and tunable P(St-*co*-GMA) porous copolymers were synthesized to immobilize *P. acidilactici* using w/o high internal phase emulsions as templates, where GMA provided anchor points for the cells to be firmly tethered to the void walls. During the fermentation of immobilized *P. acidilactici*, the open-cell structure of the polyHIPE permitted efficient substrate transfer, and the mass transfer increased along with enhanced interconnectivity of the monolith, resulting in higher _L_-lactic acid yield. In cyclic fermentation studies, the immobilized *P. acidilactici* maintained produced over 92.9% of their initial relative lactic yield production after 10 cycles, exhibiting both its great cycling stability and the durability of the material structure. The polyHIPE scaffold in this work offers tremendous potential in multiple-cell co-immobilization and cascade fermentation.

## Figures and Tables

**Table 1:** Composition of emulsion templates and properties of the resulting polyHIPEs.

Sample	Span80 (wt.%) ^[a]^	ƒ_internal phase_ (vol.%) ^[b]^	D (μm) ^[c]^	d (μm) ^[d]^	Interconnectivity (%) ^[e]^
PHI-0590	5	90	18.9 ± 5.9	3.4 ± 1.8	6.1
PHI-1090	10	90	12.5 ± 3.1	3.2 ± 1.9	12.3
PHI-2090	20	90	11.6 ± 4.9	3.6 ± 1.4	25.3
PHI-1075	10	75	16.1 ± 4.6	3.3 ± 1.4	10.5
PHI-1080	10	80	14.1 ± 3.8	3.1 ± 1.2	11.5

^[a]^ Mass fraction of Span80 with respect to monomers. ^[b]^ Internal (aqueous) phase volume fraction compared to the emulsion’s volume. ^[c]^ Average diameter of void. ^[d]^ Average diameter of interconnected pores. ^[e]^ Interconnectivity of polyHIPEs.

## Data Availability

Not applicable.

## Supplementary Materials

The following supporting information can be downloaded at: https://www.mdpi.com/article/10.3390/polym15081862/s1, Figure S1: FT-IR spectrum of P(St-*co*-GMA) polyHIPE; Figure S2: Water wettability of polyHIPE scaffolds of PHI-0590, PHI-2090, PHI-1075, and PHI-1080; Figure S3: Water contact angle (at 0.469 s) of PHI-1090 before (a) and after (b) immobilizing *P. acidilactici*; Figure S4: ^1^H-NMR spectrum of _L_-lactic acid. The unimodal at δ = 2.50 is corresponding to DMSO-d6 solvent; Figure S5: SEM image of *P. acidilactici* immobilized PHI-1090 after 10 batches of fermentation. The inset is the corresponding PHI photographs; Figure S6: Photograph of *P. acidilactici* immobilized PHI scaffold fermenting in a fermenter; Table S1: _L_-lactic acid yield by suspended *P. acidilactici* and *P. acidilactici* immobilized PHI-1090; Table S2: _L_-lactic acid yield by *P. acidilactici* immobilized on polyHIPEs with different structure; Table S3: _L_-lactic acid yield enhancement using other scaffolds for cell immobilization; Table S4: _L_-lactic acid yield and relative lactic acid yield of suspended *P. acidilactici.*
