# Supplementary material for: Hierarchical Emulsion-Templated Monoliths (polyHIPEs) as Scaffolds for Covalent Immobilization of P. acidilactici
Source: Polymers (Basel). 2023 Apr 13;15(8):1862. doi: 10.3390/polym15081862 (PMC10145616; doi:10.3390/polym15081862)
Supplement: Supplementary file 1 [file polymers-15-01862-s001.zip › polymers-2303324-supplementary.pdf]

Supporting information

Hierarchical emulsion-tempalted monoliths (polyHIPEs) as scaffolds for covalent immobilization of P. acidilactici

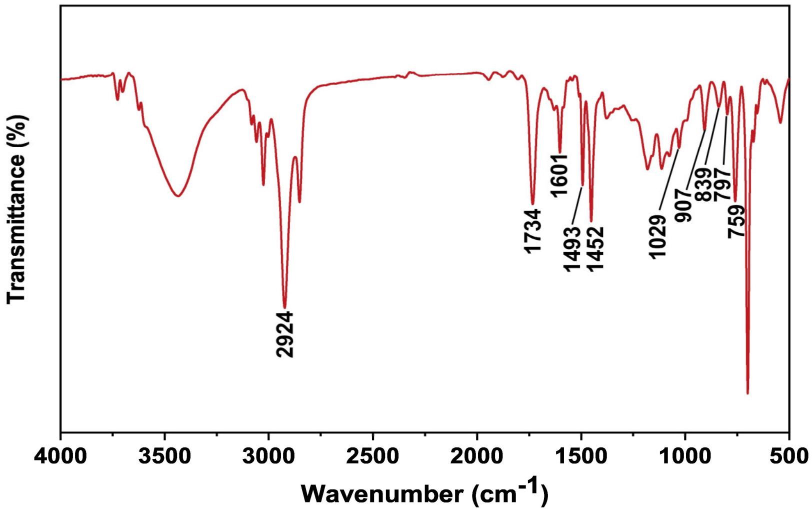

Figure S1. FT-IR spectrum of P(St-*co*-GMA) polyHIPE.

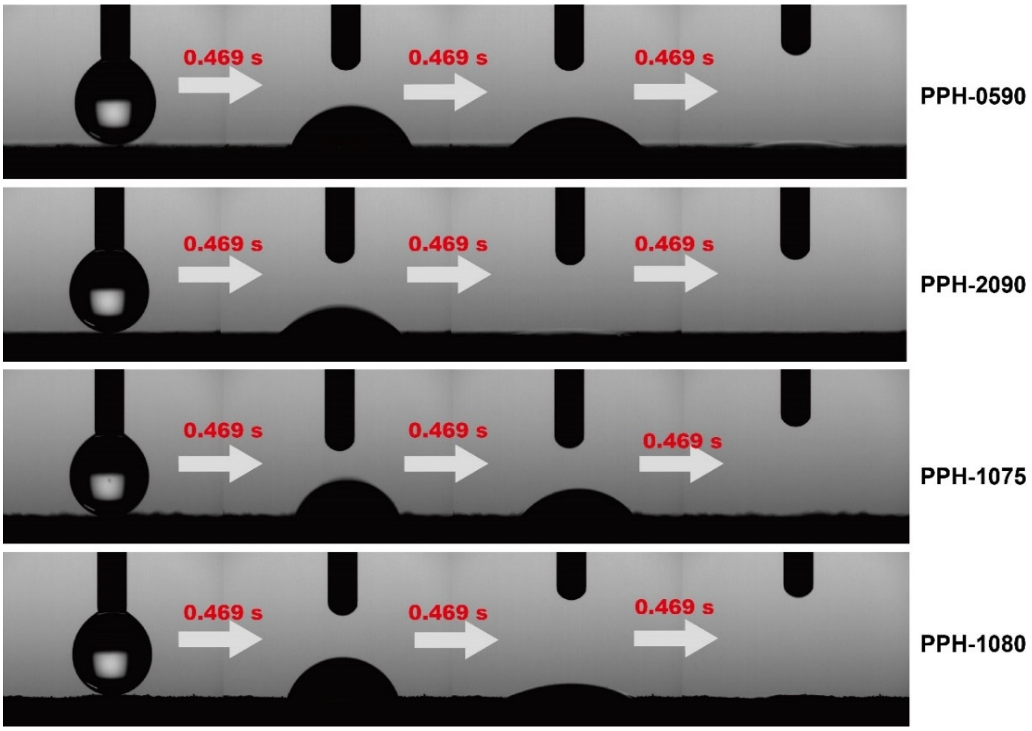

Figure S2. Water wettability of polyHIPE scaffolds of PHI-0590, PHI-2090, PHI-1075, PHI-1080.

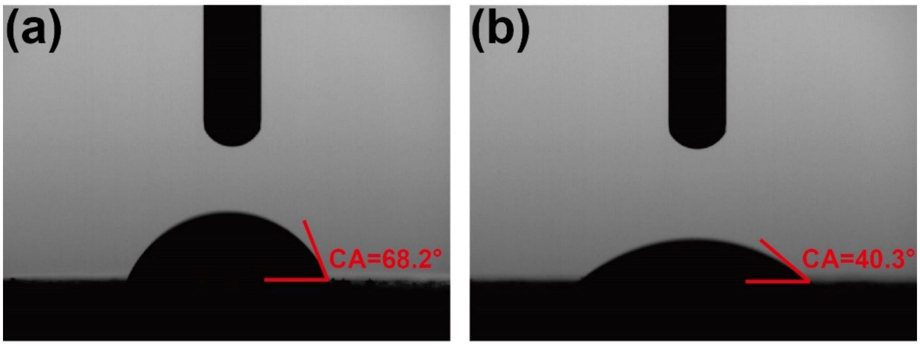

Figure S3. Water contact angle (at 0.469 s) of PHI-1090 before (a) and after (b) immobilizing *P. acidilactici*.

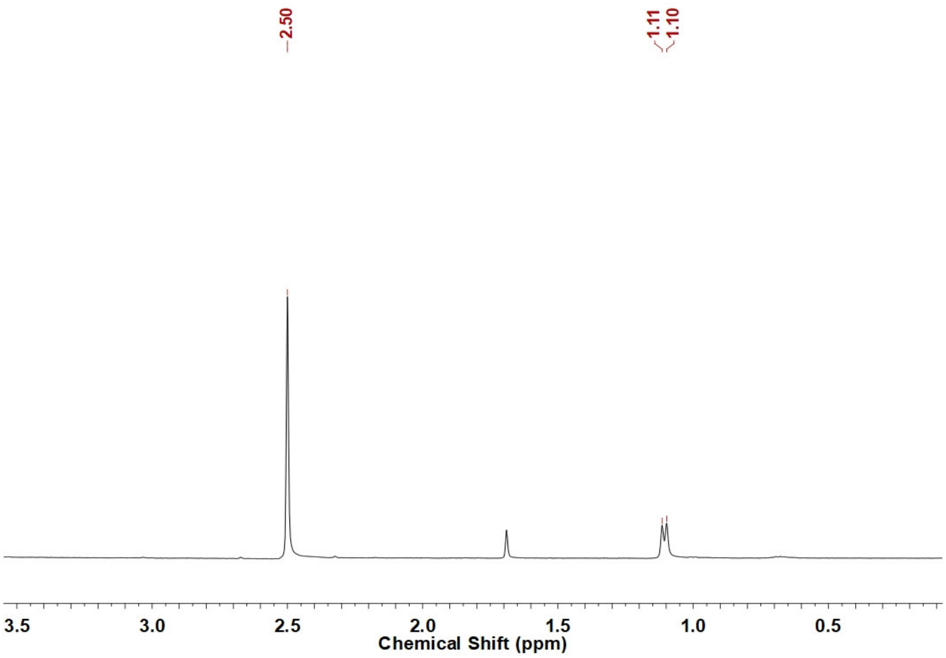

Figure S4. ^{1}H-NMR spectrum of L-lactic acid. The unimodal at δ = 2.50 is corresponding to DMSO-d6 solvent.

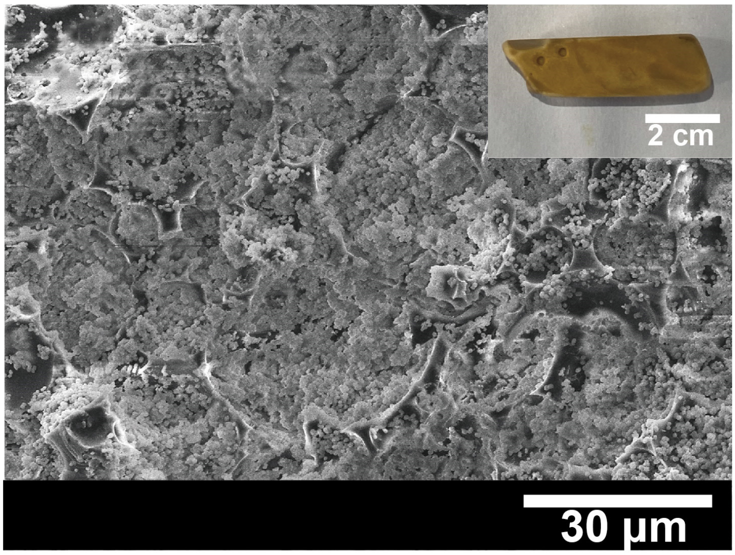

Figure S5. SEM image of *P. acidilactici* immobilized PHI-1090 after 10 batches fermentation. The inset is the corresponding PHI photographs.

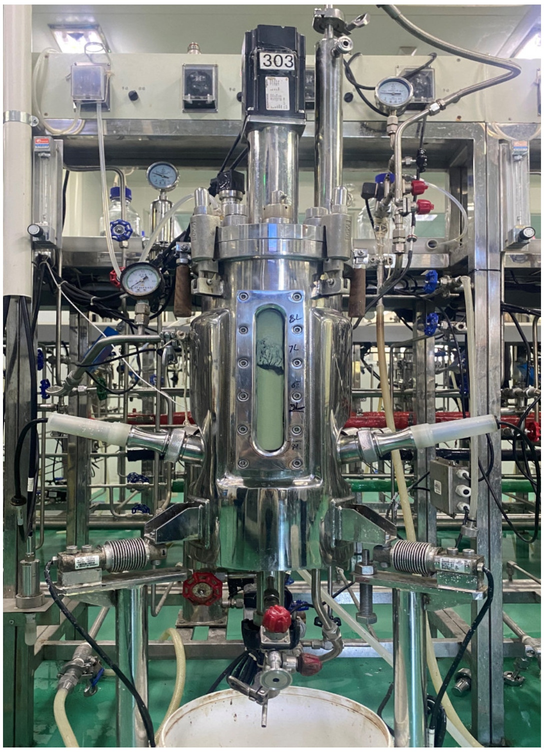

Figure S6. Photograph of *P. acidilactici* immobilized PHI scaffold fermenting in a fermenter.

Table S1. L-lactic acid yield by suspended *P. acidilactici* and *P. acidilactici* immobilized PHI-1090.

**Temperature**	**L-lactic acid of suspended *P. acidilactici* (g/kg) [a]**	**L-lactic acid of immobilized *P. acidilactici* (g/kg) [a]**
38 ℃	26.1±0.9	32.0±1.1
40 ℃	28.0±0.7	28.7±1.2
42 ℃	29.1±1.5	34.0±1.0
44 ℃	23.8±0.9	24.3±0.6
46 ℃	26.6±0.8	27.6±1.0
48 ℃	27.7±1.4	28.1±0.6

[a] Ratio of L-lactic acid mass to fermentation medium weight.

Table S2. L-lactic acid yield by *P. acidilactici* immobilized on polyHIPEs with different structure.

**Sample**	**L-lactic acid of immobilized *P. acidilactici* (g/kg) [a]**
Suspended *P. acidilactici*	29.1±1.2
PHI-0590	29.6±0.7
PHI-1090	34.0±1.0
PHI-2090	28.9±1.5
PHI-1075	31.7±0.8
PHI-1080	32.1±0.9

[a] Ratio of L-lactic acid mass to fermentation medium weight.

Table S3. L-lactic acid yield enhancement using other scaffolds for cell immobilization.

**Scaffold**	**Biocatalyst**	**L-lactic acid yield enhancement [a]**	**References**
SA-PVA gel	*Lactobacillus pentosus*	11.3%	[1]
Apple pomace	*Pediococcus acidilactici*	15.3%	[2]
Mesoporous silica-based material	*Lactobacillus rhamnosus*	4.1%	[3]
Microtube array membrane	*Lactobacillus acidophilus*	7.2%	[4]

[a] Increased percentage of L-lactic acid yield by immobilized cells compared to free cells.

Table S4. L-lactic acid yield and relative lactic acid yield of suspended *P. acidilactici*

Cycle number	L-lactic acid yield by suspended P. acidilactici (g/kg) [a]	Relative lactic acid yield by suspended P. acidilactici (%)
1	30.7±0.2	100
2	28.8±1.3	93.5
3	28.2±0.8	91.8
4	27.6±1.5	89.9
5	28.9±1.7	93.9
6	24.8±0.9	80.8
7	30.3±1.6	98.5
8	29.5±0.8	95.9
9	29.6±1.6	96.4
10	28.7±1.9	93.3

[a] Ratio of L-lactic acid mass to fermentation medium weight.

Reference

1. Wang, J.; Huang, J.; Guo, H.; Jiang, S.; Zhang, J.; Ning, Y.; Fang, M.; Liu, S. Optimization of immobilization conditions for Lactobacillus pentosus cells. *Bioprocess Biosyst. Eng.* **2020**, 43, 1071-1079, doi:10.1007/s00449-020-02305-9.
2. Bartkiene, E.; Vizbickiene, D.; Bartkevics, V.; Pugajeva, I.; Krungleviciute, V.; Zadeike, D.; Zavistanaviciute, P.; Juodeikiene, G. Application of Pediococcus acidilactici LUHS29 immobilized in apple pomace matrix for high value wheat-barley sourdough bread. *LWT Food Sci. Technol.* **2017**, 83, 157-164, doi:10.1016/j.lwt.2017.05.010.
3. Zhao, Z.; Xie, X.; Wang, Z.; Tao, Y.; Niu, X.; Huang, X.; Liu, L.; Li, Z. Immobilization of Lactobacillus rhamnosus in mesoporous silica-based material: An efficiency continuous cell-recycle fermentation system for lactic acid production. *J. Biosci. Bioeng.* **2016**, 121, 645-651, doi:10.1016/j.jbiosc.2015.11.010.
4. Chen, C.-C.; Lan, C.-C.; Pan, C.-L.; Huang, M.-Y.; Chew, C.-H.; Hung, C.-C.; Chen, P.-H.; Lin, H.-T.V. Repeated-batch lactic acid fermentation using a novel bacterial immobilization technique based on a microtube array membrane. *Process Biochem.* **2019**, 87, 25-32, doi:10.1016/j.procbio.2019.09.016.
